# Extracorporeal life support for management of refractory cardiac or respiratory failure: initial experience in a tertiary centre

**DOI:** 10.1186/1757-7241-18-28

**Published:** 2010-05-21

**Authors:** Adriano Peris, Giovanni Cianchi, Simona Biondi, Manuela Bonizzoli, Andrea Pasquini, Massimo Bonacchi, Marco Ciapetti, Giovanni Zagli, Simona Bacci, Chiara Lazzeri, Pasquale Bernardo, Erminia Mascitelli, Guido Sani, Gian Franco Gensini

**Affiliations:** 1 Anaesthesia and Intensive Care Unit of Emergency Department, Careggi Teaching Hospital, Florence, Italy; 2 Postgraduate School of Anesthesia and Intensive Care, Faculty of Medicine, University of Florence, Italy; 3 Cardiac Surgery, Heart and Vessel Department, Careggi Teaching Hospital, Florence, Italy; 4 Intensive Cardiac Coronary Unit, Heart and Vessel Department, Careggi Teaching Hospital, Florence, Italy

## Abstract

**Introduction:**

Extracorporeal Life Support (ECLS) and extracorporeal membrane oxygenation (ECMO) have been indicated as treatment for acute respiratory and/or cardiac failure. Here we describe our first year experience of in-hospital ECLS activity, the operative algorithm and the protocol for centralization of adult patients from district hospitals.

**Methods:**

At a tertiary referral trauma center (Careggi Teaching Hospital, Florence, Italy), an ECLS program was developed from 2008 by the Emergency Department and Heart and Vessel Department ICUs. The ECLS team consists of an intensivist, a cardiac surgeon, a cardiologist and a perfusionist, all trained in ECLS technique. ECMO support was applied in case of severe acute respiratory distress syndrome (ARDS) not responsive to conventional treatments. The use of veno-arterial (V-A) ECLS for cardiac support was reserved for cases of cardiac shock refractory to standard treatment and cardiac arrests not responding to conventional resuscitation.

**Results:**

A total of 21 patients were treated with ECLS during the first year of activity. Among them, 13 received ECMO for ARDS (5 H1N1-virus related), with a 62% survival. In one case of post-traumatic ARDS, V-A ECLS support permitted multiple organ donation after cerebral death was confirmed. Patients treated with V-A ECLS due to cardiogenic shock (N = 4) had a survival rate of 50%. No patients on V-A ECLS support after cardiac arrest survived (N = 4).

**Conclusions:**

In our centre, an ECLS Service was instituted over a relatively limited period of time. A strict collaboration between different specialists can be regarded as a key feature to efficiently implement the process.

## Introduction

Extracorporeal circulation support techniques have been proposed either for treatment of cardiac and/or pulmonary failure refractory to conventional treatments in adult patients. The first device, which assured blood extracorporeal oxygenation and perfusion of isolated organs, was developed by von Frey and Gruber in 1885 [1]. The first heart-lung machine was projected by Gibbon in 1937 in order to allow open-heart surgical operations [2]. Over the years, extracorporeal circulation circuit has been improved and the technique optimized, and it is now available for clinical practice. From a general point of view, two methods of support are outlined: veno-venous extracorporeal oxygenation, commonly known as ECMO, Extracorporeal Membrane Oxygenation, for respiratory function substitution and extracorporeal life support technique (ECLS) with a veno-arterial circulation for both oxygenation and hemodynamic assistance. The major indications for ECMO, in adult patients, are severe acute respiratory distress syndrome (ARDS) refractory to conventional treatments [3,4], and, in selected cases, post-traumatic respiratory failure, severe asthma [5,6], and chronic lung disease waiting for lung transplantation [7,8].

The indications for ECLS and cardiac support are cardiac failure due to any cause, and cardiac arrest not responsive to Advanced Life Support manoeuvres. In the first case, the in-hospital mortality rate is still high (between 33% and 38%) and ECLS represents a rescue-therapy useful for refractory patients [9]. In case of in-hospital cardiac arrest, when ECLS was used after ten minutes of unsuccessful cardiopulmonary resuscitation, an increase in survival rate at ICU discharge, at 30-day and at 1-year survival was reported [10].

At a tertiary referral trauma center (Careggi Teaching Hospital, Florence, Italy) an ECLS program was developed beginning April 2008 by the Intensive Care Unit of Emergency Department in association with the Intensive Cardiac Coronary Unit of Heart and Vessel Department. Here we describe our experience in implementing a multidisciplinary ECLS team for cardiac and respiratory failure. In addition to reporting our clinical experience, we present the algorithm for ECLS activation for in-hospital cardiac arrest and the experience of a national referral center for treatment of H1N1 influenza related respiratory failure.

## Methods

### ECLS Team

The ECLS team consists of an intensivist, a cardiac surgeon, a cardiologist and a perfusionist, all trained on ECLS technique and management. According to our activation protocol, ECLS team can be summoned within one hour, with 24 hour coverage.

In most cases, the intensivist primes the process on the basis of clinical and radiological findings and activates the full ECLS team. The cardiologist's main task is to evaluate cardiac function in the pre-ECLS phase and guides the correct positioning of ECLS cannulas by transesophageal ultrasonography. Furthermore, the cardiologist is directly involved in selecting patients with cardiac failure suitable for ECLS treatment. The cardiac surgeon, in addition to actively participating to the clinical decision making process, is responsible for selecting and inserting the cannulas and starting the extracorporeal circulation, with the assistance of the perfusionist.

In case of an ECLS run, irrespectively of the unit where the patient was admitted (General or Cardiac ICU), all the professionals of the team were available for consultation and performed at least one daily evaluation.

This study, supported by institutional funds only, followed the principles of the Helsinki declaration and was approved by the Internal Review Board. Informed consent for data publication was obtained.

### ECLS for respiratory failure

Veno-venous ECLS treatment (ECMO) was applied in case of severe ARDS not responsive to conventional treatments, but potentially reversible. Conditions of severe hypoxia or hypercapnia, where the limits of a protective ventilation strategy could not be maintained (tidal volume less than 6 mL/Kg of predicted body weight and plateau pressure less than 30 cmH_2_O), were the indications for starting extracorporeal circulation [11].

The Careggi Teaching Hospital had started a collaboration with the ICUs of 12 district hospitals in Tuscany in a pilot project for centralization of acute lung injury/ARDS patients who require (or may require) ECLS treatment. In 2008 and spring 2009, preliminary meetings were organized to inform the peripheral hospitals' ICU staff and Administrations about the availability of the new ECLS program. During the H1N1 influenza A pandemic, the knowledge of ECMO treatment rapidly spread among the medical community and the Regional Ministry of Health issued indications to transfer all patients affected by severe respiratory failure related to influenza to Careggi Hospital. In Appendix 1 is reported the set of parameters that were adopted to quickly detect patients suitable for extracorporeal treatment in the peripheral hospitals.

Patients deemed suitable for ECMO treatment were evaluated on site by the ECMO team. Depending on clinical condition, the transfer was performed on conventional ventilation or, alternatively, ECLS treatment was initiated in the peripheral hospital and maintained during transportation [12].

We preferentially adopted a high flow technique (5-6 litres per minute of blood flow), to maximize the opportunity of providing protective ventilation, aiming to achieve a plateau pressure below 28 cm H_2_O and PEEP 2 cmH_2_O above the lower inflection point of the quasi-static pressure volume curve, regardless the delivered tidal volume (in any case less than 6 ml/kg). Controlled respiratory frequency was reduced to 4-10/min to maintain normocapnia. Inspired oxygen fraction was reduced to 0.5 or lower, whenever possible. A recruitment manoeuvre was performed at least once a day, and ventilation with an intermittent high pressure breath ("sigh") was adopted to improve lung aeration [13]. During ECMO, nitric oxide administration [14], vasoactive support, and prone positioning were maintained or initiated according to clinical conditions.

### ECLS for hemodynamic support

The use of ECLS for cardiac support was reserved for cases of cardiac shock refractory to standard treatments and cardiac arrests not responding to conventional resuscitation. According to our internal protocol, ECLS was adopted also as a bridge to implantation of Left Ventricular Assist Device or to heart transplantation [15].

ECLS was employed in cases of in-hospital cardiac arrest when the patient was considered to have a good chance of recovery both for clinical conditions and for the timing of resuscitation. An age limit of seventy years, severe irreversible brain damage, terminal malignancy, pre-signed "do not attempt resuscitation" orders and contraindications to prolonged systemic heparin infusion were the only strict exclusion criteria taken into account. In case of cardiac arrest, hypothermia was rapidly initiated and was maintained for 24 hours at a temperature between 32-34°C [16].

Veno-arterial (V-A) ECLS treatment was considered contraindicated, when a severe aortic incompetence, aortic dissection or ventricular thrombosis was detected by echocardiography.

### Equipment

The ECLS circuit consisted of a Rotaflow Maquet Centrifugal Pump (Maquet, Rastatt, Germany) and a hollow fiber membrane oxygenator (Quadrox-D Oxygenator, Maquet, Rastatt, Germany), connected with biocoated tubes. In the V-A circuit, blood was drained through femoral vein and reinfused into aorta through femoral artery. For V-V ECLS two types of cannulas were used. At the beginning, Raumedic cannulas ranging from 21 to 28 french (Raumedic AG, Germany) were employed with femoral and jugular vein cannulation. Since July 2009, Avalon Elite™ Bi-Caval Dual Lumen Catheters have become available. These specially designed dual lumen cannulas, inserted in the right internal jugular vein, permit both drainage and reinfusion of blood. In V-A ECLS, the distal perfusion of the limb could be jeopardized by the relatively large bore inflow cannula, inserted in the femoral artery at the groin: to prevent leg ischemia, we usually inserted a small shunt cannula (14 french) in the femoral artery, distally to the ECLS cannula. Heparin therapy was titrated by bedside measurement of activated partial thromboplastin time (aPTT) with Hemochron (Hemochron Jr. Sign. plus, ITC Europe, Milan, IT) every two hours.

Numerical data were summarised as median and interquartile range.

## Results

A total of 21 patients were treated with ECLS during the first one year of activity (April 2008 - December 2009). Among them, 13 were treated with ECMO for respiratory failure (Table 1), and 8 were treated with V-A ECLS due to cardiac arrest (Table 2) and cardiogenic shock (Table 3). The most frequent complication observed was local bleeding from the insertion points of the cannulae, central line access site and tracheostomy (36%). In one case, oxygenator failure occurred due to clots formation; in this occasion a rapid increase of D-Dimers was observed, followed by a worsening of oxygenation and decarboxylation performance of the artificial lung. Circuit change was promptly carried out with no further complications. In one case of V-A ECLS, major bleeding occurred at site of cannulae insertion several days after successful weaning, requiring multiple transfusions. At surgical inspection a femoral artery wall lesion was found and required prosthetic repair.

**Table 1:** Patients treated with ECMO for respiratory failure.

Patients	Age/Gender	SAPS II at admission	Indication	Time to ECMO from ARDS diagnosis (days)	Hours of critical hypoxia (PaO_2_/FiO_2 _< 60) and/or critical acidosis (pH < 7.20)	ECMO duration (hours)	ECMO-related complications	ICU LOS (days)	ICU outcome
Pt 1	70/M	27	ARDS (infective)	12	4	269	Local bleeding	33	Survived

Pt 2	19/M	47	ARDS (post-traumatic)	1	4	33	None	3	Non survived

Pt 3	64/F	62	ARDS (infective)	1	2	44	Intracranial bleeding	3	Non survived

Pt 4	65/M	58	ARDS (post-traumatic)	1	1	231	Local bleeding	65	Survived

Pt 5	69/M	29	ARDS (infective)	1	5	264	Local bleeding	20	Survived

Pt 6	67/M	82	ARDS (infective)	10	8	253	Local bleeding	12	Non survived

Pt 7	59/M	50	ARDS (infective)	4	8	626	Oxygenator failure	27	Non survived

Pt 8	61/M	62	ARDS (infective)	5	5	330	None	15	Non survived

Pt 9	15/M	66	ARDS (H1N1)	0	5	137	None	17	Survived

Pt 10	58/M	24	ARDS (H1N1)	2	4	154	None	13	Survived

Pt 11	44/F	46	Viral acute lung failure (H1N1)	2	5	374	Local bleeding	20	Survived

Pt 12	48/M	40	ARDS (H1N1)	1	6	184	Local bleeding	17	Survived

Pt 13	30/M	44	Viral acute lung failure (H1N1)	1	6	151	None	18	Survived

**Table 2:** Patients treated with V-A ECLS for in-hospital cardiac arrest.

Patients	Age/Gender	SAPS II at admission	Diagnosis at hospital admission	Cardiac arrest etiology	Initial rhythm of cardiac arrest	ACLS duration to ECLS (minutes)	Return of Spontaneous Circulation (ROSC)	ECLS duration (hours)	ECLS-related complications	ICU LOS (days)	ICU outcome
Pt 1	66/F	60	Septic shock	Multi Organ Dysfunction Syndrome	Asystole	60	No	6	None	2	Non survived

Pt 2	59/M	69	Cardiac arrest	Bridge to diagnosis	Pulseless electrical activity	90	Yes	25	None	2	Non survived

Pt 3	16/M	40	Trauma with brain injury	Trauma	Pulseless electrical activity	45	No	46	None	9	Non survived

Pt 4	23/M	80	Trauma	Hemorrhagic shock	Pulseless electrical activity	55	No	3	None	1	Non survived

**Table 3:** Patients treated with V-A ECLS in case of cardiogenic shock.

Patients	Age/Gender	SAPS II at admission	Cardiogenic shock etiology	Intra Aortic Balloon Pump	ECLS duration (hours)	ECLS-related complications	ICU LOS (days)	ICU outcome
Pt 1	43/M	61	Post cardiac arrest heart failure-ARDS in near-drowning	Yes	120	None	26	Survived

Pt 2	55/F	67	Multi Organ Dysfunction Syndrome	No	72	None	5	Non survived

Pt 3	68/M	91	Multi Organ Dysfunction Syndrome	No	24	None	2	Non survived

Pt 4	24/F	47	Post cardiac arrest heart failure	Yes	189	Local bleeding, aneurysm	14	Survived

Five patients received renal replacement therapy (continuous veno-venous hemofiltration, CVVH). The CVVH was connected in-line to the extracorporeal circuit with the withdrawal line before oxygenator and return line after the oxygenator. Renal function recovered in all cases, and both ECLS and CVVH run was uneventful on this configuration.

During extracorporeal support, invasive procedures were carried out without any immediate complications. Among these, four bedside percutaneous tracheotomies (Ciaglia technique) were performed, and two narrow bore pleural catheters were inserted under ultrasound guidance for massive pleural effusions. Autopsy was performed in all non surviving patients and no lesion of vessels due to the presence of cannulae was observed.

## ECMO for respiratory failure

A total of 13 patients were treated with ECMO for ARDS: six patients were affected by bacterial pneumonia, five patients had H1N1-related ARDS (two with Legionella Pneumophila superinfection), and 2 patients presented trauma-related respiratory failure. Data of each patient are represented in Table 1. Median age was 59 years (IQR 44-65), with a prevalence of male sex (85%). Median ICU length of stay was 17 days (IQR 13-20). Eight out of 13 patients were successfully weaned from ECMO and discharged from ICU (overall survival rate of 62%). All H1N1 patients were discharged from ICU and from hospital.

The median duration of ECMO was 235 hours (IQR 151-269), with a difference between survivors (221 hours) and non survivors (257 hours). We considered time from verification of ECMO criteria to extracorporeal support start as an efficiency parameter ("time to ECMO"), and it was 6 hours (IQR 4-9).

From October 2009, when our ECMO Service became the referral centre of Central Italy for H1N1-induced ARDS, extracorporeal support was initiated in the peripheral hospital in 3 cases. Inter-hospital transport was safely performed on extracorporeal support and all patients were discharged alive from ICU.

One young patient (19 years) died due to severe traumatic brain injury. In this patient, ECMO was maintained in the first 12 hours without systemic heparin infusion and no complications occurred during extracorporeal treatment. After cerebral death confirmation, multiple organ donation was accomplished. One patient (a 64 year-old woman) died due to subarachnoidal hemorrhage, although coagulation parameters were normal.

### V-A ECLS for cardiac arrest and cardiogenic shock

Four victims of intra-hospital cardiac arrest received V-A ECLS for cardiac support (Table 2). Patients were 41 years old (median, IQR 21-61; male sex 75%). The median duration of advanced cardiac life support manoeuvres before ECLS start was 58 minutes (IQR 53-68), and the median duration of ECLS was 16 hours (5-30). In two patients, the ECLS support started in the Emergency Room. All four patients died during their ICU stay (one patient after ECLS withdrawal).

Data of the four patients treated for cardiogenic shock are represented in Table 3. Median age was 49 years (IQR 38-58, male sex 50%). Among them, 2 patients survived and were discharged from ICU. In these patients, median duration of ECLS was 96 hours (IQR 60-137). Intraaortic balloon pump was necessary in all four patients. Survival rate was 50%.

## Discussion

From the experience here reported, we can state that, with a close cooperation between different specialists (intensivist, cardiologist, cardiac surgeon, nurse, perfusionist), an ECLS Service can be started over a relatively limited period of time, achieving a high level of efficiency. Our model of ECLS team has allowed us to start extracorporeal support in different hospital scenarios, such as ICU and Emergency Room. This feature of flexibility and adaptability of our ECMO system has made it particularly beneficial during the Influenza A pandemic, making this resource available also in peripheral hospitals.

The management of a patient on ECLS is still challenging in terms of utilization of resources and commitment of health personnel. Beyond the insertion procedure, a multidisciplinary team can better accomplish the tasks of daily management of the patient, as an intensivist, a cardiac surgeon and a perfusionist should repeatedly evaluate the circuit and the patient to guarantee a safe and uneventful treatment. Furthermore, every ECLS patient needs a dedicated nurse. With the assistance of these dedicated professionals, also in-hospital transportation can be safely carried out (i.e. to radiological suite). In our population 63% of patients received a CT scan during ECLS treatment, and no transport-related complications occurred. The most common complication was local bleeding, usually simple to manage. In this regard, the use of Bioline surface-heparinized circuits allowed a limited dose of heparin, and may have reduced the incidence of complications such as coagulation, complement activation, thrombus formation and the need for transfusions [17-19].

The survival rate of 62% of our patients treated with ECMO for respiratory failure is comparable to other published studies. In 2004, Hemmila and co-workers retrospectively reviewed 255 patients with ARDS treated with ECMO between 1989 and 2004, showing a 67% of patients successfully weaned from ECMO and a hospital discharge of 52% [20]. More recently, the CESAR (Conventional ventilation or ECMO for Severe Adult Respiratory Failure) trial has shown an increase of survival rate, without severe disability, 6 months after randomization in patients treated with ECMO in comparison to conventional ventilation (63% vs 47%) [4].

From the first phase of implementation, our service was conceived to provide extracorporeal support even in peripheral institutions, therefore a dedicated ambulance was specifically prepared and all equipment arranged for transportation. In our opinion, this is a key feature for an effective ECMO service as inter-hospital transportation of patients with severe respiratory failure can be challenging due to the fact that limited possibilities of intervention are available and clinical deterioration may occur [21]. Therefore, several centres recommend the start of extracorporeal assistance before transfer [22,12]. In our out-of-hospital ECMO experience, one patient was safety transferred by ambulance from a distance of 400 Km.

We report quite a short time to establish V-A ECLS in case of in-hospital cardiac arrest (58 min). Furthermore, there is a trend towards a progressive reduction of this interval over time. Despite this remarkable performance of our ECLS system in terms of speed of response, no patient receiving extracorporeal support for cardiac arrest survived. In a large series of patients on extracorporeal support for in hospital cardiac arrest, Jaski and co-workers reported a long term survival rate of 23% in witnessed events and no survival in non-witnessed arrest. At multivariate analysis cardiac arrest in the critical care unit was found to be the only independent variable predictive of outcome [23]. In another series of 40 in-hospital cardiac arrest victims, time before ECLS was 105 minutes, and 20% survival rate was reported [24]. In our experience, the number of cardiac arrest patients with ECLS is so limited that comparison to published data is not feasible. Nevertheless, the reason for not responding to V-A ECLS treatment in our cases might be possibly related to the severity of previous clinical condition (2 traumas, 1 septic shock) and to the underling organ dysfunction.

## Conclusions

ECMO and V-A ECLS might be considered a therapeutic option in patients with severe ARDS and/or with cardiac failure or cardiac arrest. In our experience, a well-timed start of ECMO in case of ARDS, prevents the progression of ventilator-induced lung injury and increases the chances of lung recovery. Also in case of cardiogenic shock, an extracorporeal technique seems a viable option and increases the possibility of early cardiac recovery avoiding neurological damages and multi-organ failure. To guarantee a safe treatment, the involvement of several properly trained physicians and nurses seems advisable.

## Key messages

• An ECLS Service can be effectively organized in a Center were the needed competencies are available (intensivist, cardiologist, cardiac surgeon).

• When physicians and nurses are skilled in the technique, the Service can provide a safe transfer of critically ill patients from remote hospitals.

• ECMO should be considered in the initial phase of ARDS, when failure to ventilation strategy occurs.

• The resource of ECMO has resulted to be particularly important in the event of cases of severe respiratory failure, as in the last pandemic of Influenza A.

## Abbreviations

ARDS: adult respiratory distress syndrome; ECLS: extracorporeal life support; ECMO: extracorporeal membrane oxygenation; ICU: intensive care unit; PEEP: positive end-expiratory pressure; SAPS: simplified acute physiology score; TEE: transesophageal echocardiography.

## Competing interests

The authors declare that they have no competing interests.

## Authors' contributions

AP (Adriano Peris), MB, GC, AP (Andrea Pasquini), GS, GFG organized the ECLS/ECMO Service. MB (Manuela Bonizzoli), GC, AP, SB, PB, CL reviewed the literature. SB, GC, GZ, AP wrote the draft of article. SB collected data. MB (Massimo Bonacchi) performed all ECLS/ECMO insertion procedures. CL and PB performed TEE assistance. AP (Adriano Peris), MB, GC, AP (Andrea Pasquini), MC, SB and EM managed cases here reported. All Authors have seen and approved the final revised version.

## Appendix 1: First contact criteria to discuss the need of ECLS

The parameter are referred to a condition of lung protective ventilation's (tidal volume:4-6 ml/Kg of predicted body weight; plateau pressure ≤ 30 cmH_2_O; PEEP > lower inflection point of the curve pressure-volume).

PEEP: positive end-expiratory pressure; PaO_2_: arterial oxygen partial pressure; FiO_2_: inspired oxygen fraction; RR: respiratory rate; SaO_2_: peripheral oxygen saturation; SvO_2_: central venous oxygen saturation.

Acute respiratory failure with 1 of the following condition:

1. SaO_2 _< 85% for at least 1 hour

2. Oxygenation Index^1 ^>25 for at least 6 hours after ventilation's optimization

3. PaO_2_/FiO_2 _< 100 with PEEP ≥ 10 cmH_2_O for at least 6 hours after ventilation's optimization

4. Hypercarbia with pH < 7.25

5. SvO_2 _< 65% with hematocrit >30 and under vasoactive drugs infusion

^1^Mean airway pressure (cmH_2_O) * FiO_2 _* 100/PaO_2_
